# Innovations in Genomics and Big Data Analytics for Personalized Medicine and Health Care: A Review

**DOI:** 10.3390/ijms23094645

**Published:** 2022-04-22

**Authors:** Mubashir Hassan, Faryal Mehwish Awan, Anam Naz, Enrique J. deAndrés-Galiana, Oscar Alvarez, Ana Cernea, Lucas Fernández-Brillet, Juan Luis Fernández-Martínez, Andrzej Kloczkowski

**Affiliations:** 1 Institute of Molecular Biology and Biotechnology (IMBB), The University of Lahore (UOL), Lahore 54590, Pakistan; anam.naz@imbb.uol.edu.pk; 2 The Steve and Cindy Rasmussen Institute for Genomic Medicine, Nationwide Children’s Hospital, Columbus, OH 43205, USA; 3 Department of Medical Lab Technology, The University of Haripur, Haripur 22620, Pakistan; faryal_mehwish@yahoo.com; 4 Group of Inverse Problems, Optimization and Machine Learning, University of Oviedo, 33003 Oviedo, Spain; andresenrique@uniovi.es (E.J.d.-G.); jlfm@uniovi.es (J.L.F.-M.); 5 DeepBioInsights, 38311 La Florida, Spain; uo217123@uniovi.es (O.A.); cerneadoina@uniovi.es (A.C.); jlfmuniovi@gmail.com (L.F.-B.); 6 Department of Pediatrics, The Ohio State University College of Medicine, Columbus, OH 43205, USA

**Keywords:** genomics, big data analytics, personalized medicine, health, computational approaches

## Abstract

Big data in health care is a fast-growing field and a new paradigm that is transforming case-based studies to large-scale, data-driven research. As big data is dependent on the advancement of new data standards, technology, and relevant research, the future development of big data applications holds foreseeable promise in the modern day health care revolution. Enormously large, rapidly growing collections of biomedical omics-data (genomics, proteomics, transcriptomics, metabolomics, glycomics, etc.) and clinical data create major challenges and opportunities for their analysis and interpretation and open new computational gateways to address these issues. The design of new robust algorithms that are most suitable to properly analyze this big data by taking into account individual variability in genes has enabled the creation of precision (personalized) medicine. We reviewed and highlighted the significance of big data analytics for personalized medicine and health care by focusing mostly on machine learning perspectives on personalized medicine, genomic data models with respect to personalized medicine, the application of data mining algorithms for personalized medicine as well as the challenges we are facing right now in big data analytics.

## 1. Introduction

Personalized medicine is an evolving field of science aimed in using various diagnostic tests to determine which medical treatments will work best for each individual patient. The progress of personalized medicine over the ages can be characterized by several milestones.

1. More than two and a half millennia ago, Hippocrates stated: “every human is distinct, and this affects both the disease prediction and the treatment”.
2. In 1956, “favism”, the genetic basis for the selective toxicity of fava beans, was discovered to be due to a deficiency in the metabolic enzyme G6PD.
3. In 1985, Renato Dulbecco realized that, in order to advance cancer research, it was necessary to sequence the human genome.
4. In 1988, Genentech Inc. sequenced the entire human growth hormone locus (a world record), making evident the feasibility of sequencing the human genome.
5. In 1990, the Human Genome Project (HGP) was launched, and the first draft was published in 2001, with its final version in 2003.
6. Since the early 1990s, individualized treatments tailored to the genome of each patient have been envisioned but rarely realized.
7. In 1994, a diagnostic test for the prediction of the success of rHGH replacement therapy was developed, being the earliest registry of a companion molecular diagnostics (CMDx) test ever invented.
8. In 1998, when the FDA approved Herceptin (anti-EGFR mAb for EGFR+ breast tumors) and HerceptTest (to detect such tumors), it became the first “official” CMDx invented. Since then, a growing list of diagnostic packages/personalized medicine therapies has received, from the FDA, labels recognizing and recommending them.

The human genome is basically the foundation of personalized medicine, which is considered as the next generation of diagnosis and treatment. This review describes the progress of personalized medicine over time, emphasizing the important milestones achieved through time. Starting from the treatment of malaria, as the first more personalized therapeutic approach, it highlights the need for new diagnostic tools and therapeutic regimens based on the individual’s genetic background. Cutting-edge biochemical advances including single-nucleotide polymorphisms (SNPs), genotyping, and biochips have made personalized medicine a reality, justifying the use of the terminology in the last few decades. Variations such as SNPs, insertions and deletions, structural variants, and copy number variations in the human genome play a distinctive role in the manifestation and progression of diseases such as cancer, diabetes, and neurodegenerative and cardiovascular diseases. Hence, biomarkers are being investigated as a way of predicting certain diseases and also to identify patient subgroups that respond only to specific drugs. The discovery of the association between antimalarial drugs and G6PD deficiency has opened up a new perspective regarding the adverse effects of these drugs as well as a more personalized approach to the disease. This was one of the first examples that led to a big step toward the application of a more personalized therapy, which was established as a term many years later in 1991 and is currently still quite limited. Since that time, several clinical trials have proven the efficacy of trastuzumab, also resulting in establishing routine HER-2 testing in breast cancer patients and dramatically changing the therapeutic approach to those carrying the mutation. This gene is a great milestone in applied personalized medicine, clearly showing that the right choice of a drug, based on the genetic background of a patient, can have positive effects on their life.

Massive accumulation of large-scale molecular and clinical data in recent decades has radically changed personalized medicine and has raised great expectations concerning its impact on biomedical research and health care [1,2]. Personalized medicine is a practice of medicine that uses an individual’s genetic profile to guide decisions made regarding the prevention, diagnosis, and treatment of disease [3]. Personalized or precision medicine is an emerging medical practice based on a data-driven approach that considers relevant medical, genetic, behavioral, and environmental information about an individual to determine patient-specific therapy [2,4,5]. By linking together diverse datasets to reveal hitherto-unknown casual pathways and correlations, big data allows for far more precision and tailoring than was ever before possible [4]. Recent scientific advancements in high-throughput, high-resolution data-generating technologies enables cost-effective analysis of big datasets on individual health [6]. However, to analyze and integrate such large information, there is a need for new computational approaches such as faster, more integrated processors, larger computer memories, improved sensors, new much sophisticated algorithms, methodologies and cloud computing, which may guide future clinical practice by providing clinically useful information [6,7]. The development of big data approaches has enhanced the ability to probe which parts of biology may have functional and dysfunctional activity. The basic aim of precision medicine is to support the practicing clinician by making that information of pragmatic value. Precision medicine can be succinctly defined as an approach to provide the right treatments to the right patients at the right time [8]. However, for most clinical problems, precision strategies remain aspirational. The challenge of reducing biology to its component parts, then identifying which can and should be measured to choose an optimal intervention, the patient population that will benefit, and when they will benefit most, cannot be overstated. However, the increasing use of hypothesis-free, big data approaches promises to help us reach this aspirational goal [9].

This review article will offer an overview on recent advancements and an update on important developments in the analysis of big data and future strategies for personalized medicine. Technical and methodological approaches have been systemically discussed elsewhere and we direct the reader to these excellent reviews [10]. Here, we identify key conceptual and infrastructural challenges and provide a perspective on how advances can be and are being used to arrive at precision medicine strategies with specific examples [9].

## 2. The Conceptualization of Big Data

**Figure 1:**
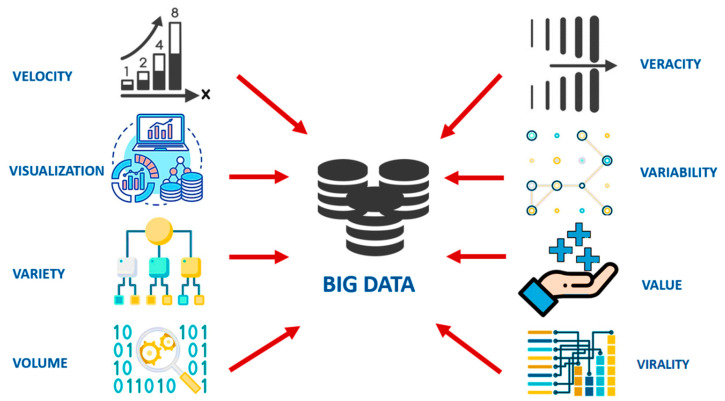
Representation of distinct dimensions of big data.

Distinct dimensions are included in the definition of “big data”, namely, volume, velocity, variety, value, variability, visualization, virality, and veracity, which describes the massive volume of structured, semi-structured, and unstructured data (Figure 1) [11,12,13,14]. According to the Health Directorate of the Directorate-General for Research and Innovation of the European Commission, big data can be defined as “Big data in health encompasses high volume, high diversity biological, clinical, environmental, and lifestyle information collected from single individuals to large cohorts, in relation to their health and wellness status, at one or several time points” [15]. Various sources of big data in the health care industry and in biomedical research include medical records of patients, results of medical examinations, and hospital records, etc. [16]. In addition, advances in technology have already created and continue to create thousands or even millions of measurements that include the sequencing of DNA, RNA, and the characterization of proteins: their sequence, structure, posttranslational modifications, and function, alongside their clinical features. In order to extract useful information from this huge amount of data, high-end computing solutions, along with appropriate infrastructure to systematically generate and analyze big data, are urgently needed. Moreover, advanced machine learning algorithms and techniques (such as deep learning, and cognitive computing) represent the future toolbox and emerging reality, which can be effectively applied to deliver integrative solutions for multi-view big data analysis in order to explain an event or predict an outcome [2]. 

Despite the recent advancements in machine learning-based solutions for big data, currently, there exist only a few examples that have considerable influence on current clinical practice. Reasons might be a lack of validation via prospective clinical trials, unsatisfactory performance of predictive models, and difficulties in interpreting complex models [16].

It is important to note that when working with genetic data, we should consider that the number of examples (patients) is usually very small in relation to the number of genes or genetic variables that are measured. Therefore, the solution is bounded by the number of patients instead of the number of variables, which makes it *a little big data* problem. This causes the uncertainty space of the mathematical models that are built to solve this kind of problems and make decisions (regressors or classifiers) to have a huge uncertainty space that contains the set of models that predict the observed data within the same error bounds. These models are located in flat curvilinear valleys of the cost function landscape [17,18]. This holds independently of the inverse problem that it is being solved and concerns the uncertainty analysis of inverse problems and classification problems, which are by definition ill-posed. In this way, these problems are very difficult to solve since the noise from the data might dramatically perturb the solution by generating spurious unphysical solutions. Therefore, the best way to deal with such problems is by reducing the dimension to perform a robust uncertainty analysis of the corresponding medical decision problem [19,20]. This kind of approach needs robust sampling methods to consider possible multiple scenarios.

Data formatting and the storing of data also remain as big challenges in the past years. However, the last decade has seen remarkable progress in the development of standard genomic data formats such as FASTQ, BAM/CRAM, and VCF files [21]. However, such standardization is incomplete and may lead to incompatibility between the inputs and outputs of different bioinformatics tools, or worse, inaccurate results. Therefore, imperfect standardization has allowed for the sharing of genomic data across institutions into either aggregated databases such as ExAC, GNOMAD [22] or as federated databases such as the Beacon Network [23]. ExAC, GNOMAD, and the Beacon Network databases provide support in the understanding of genetic variations and identifying variants that are unique within a specific ethnic group [22]. However, despite these successes with upstream genomic data formats, key challenges are still present related to downstream data formats. This often results in non-uniform analysis, and indeed, re-analysis of the same data using different pipelines yields different outcomes [24,25].

## 3. Computational Approaches toward Personalized Medicine

Personalized medicine refers to the patient’s treatment based on their personal clinical characterization [26]. The patient’s individual characteristics are used to modify treatment in a way that might be more intricate compared to the standard course [27]. It is evident from recent advances in the pharmacological and genetic behavior of various drugs that genetic variations in a single individual could lead to differences in the response to drugs [28]. All of these factors conspire with the notion of personalized medicine. The main aim of personalized medicine is to achieve the right treatments being given to the right patients.

**Figure 2:**
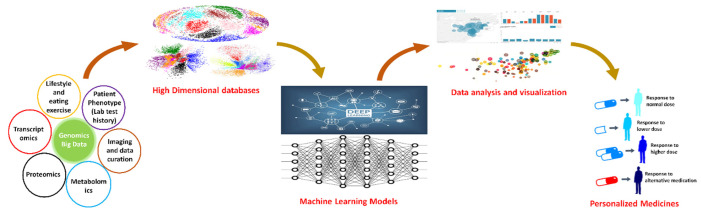
The overall computational approach for personalized medicine.

There has been a rapid development in various high-throughput technologies that has headed toward the addition of a large amount of molecular and cellular biology-related data, providing unprecedented insights into various cellular processes. These computational approaches are now exploiting these extensive data to better understand patient diagnosis, various underlying disease mechanisms, and possible treatment options (Figure 2). Based on genomic, epigenomic profiles, and drug and treatment responses, computational methods can classify the patients into different subtypes that can be helpful in disease prediction, the diagnosis of various cancers, generating disease decision rules, and personalized recommendation systems [29]. These advancements have led many research groups to investigate different aspects of personalized medicine such as diagnosis, prognosis, and pharmacogenomics through computational approaches [30]. Moreover, such approaches not only refine the existing disease maps but are also beneficial in the development of a predictive model of various diseases [29]. Such analysis is also helpful to differentiate the cellular and molecular mechanism at the normal or control state in comparison to the disease progression state. Thus, these computational approaches for personalized medicine are likely to significantly reshape the therapeutic field in the coming decades. Together, these approaches will allow for the development of various predictive models against various diseases, especially the rare ones.

Nowadays, computational models are integrated in different fields in medicine and drug development, ranging from disease modeling and biomarker research to the assessment of drug efficacy and safety [31]. The added value of such computational models, sometimes called digital evidence, in medicine is also acceptable by the scientific community [32,33] and the U.S. Food and Drug Administration (FDA) or the European Medicines Agency (EMA) [34,35]. There are two types of models: mechanistic models and data derived models. The basic aim of mechanistic models is the structural representation of the governing physiological processes in the model equations to support a functional understanding of the underlying mechanisms. On the other hand, data-driven approaches (machine learning (ML) and deep learning (DL) use algorithms and artificial intelligence (AI) methodology [36,37].

### 3.1. Molecular Interaction Maps (MIMs) 

MIMs actually represent the physical and causal interactions based on knowledge based information among biological species in the form of networks [38]. MIMs explore the information about different mechanistic pathways and regulatory modules involved in a disease such as Parkinson’s [39] or signaling in cancer [40], respectively. The basic principle of MIMs uses graph-theory concepts to identify network static properties such as (i) the identification of critical nodes; (ii) community detection; and (iii) prediction of hidden links. Furthermore, upon overlying expression data, such maps serve as visualization tools for the activity level of regulators and their targets of established disease markers, which provide the simplest mechanistic visualization of data [31].

### 3.2. Constraint-Based Models

Genome-scale metabolic (GEM) models are the best example of constraint-based models that provide a mathematical framework to understand the metabolic capacities of a cell, enabling system wide analysis of genetic perturbations, exploring metabolic diseases, and finding the essential enzymatic reactions and drug targets [41]. Most importantly, the GEM modeling approach is being used in multiple medical domains such as cancer [42] obesity [43], and in Alzheimer’s disease [44].

### 3.3. Boolean Models (BMs)

BMs are the simplest logic-based models in which nodes are assigned one of two possible states: 1 (ON, activation) or 0 (OFF, inactivation) [45]. Moreover, the regulatory relationship between regulators (upstream nodes) to targets (downstream nodes) are expressed by logical operators such as AND, OR, and NOT, respectively. Therefore, BM does not require detailed kinetic data for parameter estimation, which makes them useful for application to large biological systems. In the context of systems medicine, this approach is often applied for cancer research [46,47].

### 3.4. Quantitative Models (QMs)

QM are like ordinary differential equation (ODE)-based approaches used to analyze the quantitative behavior of a biochemical reaction with time. QMs consist of a set of differential equations containing variables and parameters that describe how the system responds to different stimuli or perturbations [48]. This quantitative modeling approach explains the biological-systems dynamics in detail and applies to a single pathway due to the requirement of detailed kinetic data for parameter estimations. Most importantly, in personalized medicine, ODE models are applied for individual biomarker discovery [49], drug response, and tailored treatments [50].

### 3.5. Pharmacokinetic Models

Pharmacokinetic models explain the concentration of a drug in plasma or different tissues. Therefore, drug pharmacokinetics are promptly used as a surrogate for drug-induced responses. Therefore, pharmacokinetic models can be described by compartmental pharmacokinetic (PK) modeling [51] or by physiologically based PK (PBPK) modeling [52].

## 4. Machine Learning Perspectives on Personalized Medicine

Machine learning imposes a major societal impact in many computational biology applications [53,54]. It has also witnessed dramatic progress as it attempts to identify patterns, rules, and many statistical dependencies in large available datasets. Nowadays, personalized medicine in relation to machine learning programs is considered as an emerging reality and is strongly connected with genomics and proteomics datasets. Machine learning approaches have been applied to massive data collected through genome sequencing, with the aim to precisely define what treatment method will work for an individual [55]. These methodologies have provided deep understanding of the underlying disease mechanisms, while integration of the assorted patient data results in amended and robust biomarker discovery for various disease diagnoses. It has been assessed that without machine learning approaches, the full potential of personalized medicine is impossible to comprehend in clinical practice. Based on machine learning approaches, various algorithms focused on specific diseases have been proposed. Among them, there is an FDA approved MammaPrint prognostic test for breast cancer based on 70 gene signatures [56]. MammaPrint is a microarray-based signature method using formalin-fixed-paraffin-embedded (FFPE) or fresh tissue for microarray analysis [57,58]. Moreover, the BluePrint test has also demonstrated the expression data, which could be supportive for personalized medicine in MINDACT and IMPACt trials [59].

Similarly, Bejnordi et al. reported an algorithm that is trained to detect metastases in various lymph nodes in stained tissue sections of breast cancer [60]. A machine learning echocardiography algorithm proposed by Madani et al. provided an accuracy of greater than 90% for the diagnosis of cardiac disease [61]. For the early detection of Alzheimer’s disease, Ding et al. proposed a machine learning based system with high accuracy and sensitivity [27].

Machine learning and AI approaches work with different types of data including genetic, genomic [62], epigenomic [63,64], transcriptomic [65], metabolomic data [66], medical images, biobanks data [67], electronic health records (EHR) [68], scientific literature data, etc., and are able to combine all of this information to design optimum classifiers [69]. In this respect, two problems including regression and classification problems are of interest. The difference between them is that in regression, the aim is to predict the value of continuous and real value quantities, for instance, to predict the level of cholesterol in blood based on other biomarkers. In the case of classification problems, the aim is to predict the label of a set of individuals that are gathered in a broad class, for instance, the patients that have a survival time greater than the average from the rest. The interest in formulating these prediction problems as classification problems comes from the reduction in the uncertainty space. Particularly, phenotype prediction problems are of great use to better understand the altered genetic pathways that are responsible for the development of the disease and to speed up the drug discovery process [70].

## 5. Modeling Genetic Data with Translational Purposes

The genetic and epigenetic regulation of the altered pathways in a cell is one of the main topics in pharmacogenomics and consists of understanding how a mutation in the DNA impacts the transcriptome and the proteome downstream [70,71,72]. Additionally, the epigenomic regulation of the transcriptome can be achieved via epigenomics through chemical compounds that bind to the DNA and alter gene expression [73]. Transcriptomics explores how gene expressions, genetic pathways, and regulatory networks are altered in each phenotype, for instance, disease vs. healthy controls. Based on these findings, it is possible to perform drug repositioning using connectivity map (CMAP) technologies provided by the Broad Institute [74]. Drug repurposing, also called drug repositioning, of the already known FDA approved compounds for new therapeutic uses is a very effective methodology to find a cure in rare diseases where the economic constraints for new drug development are very important [75]. There are multiple examples of personalized medicine being used against multiple diseases. For example, Herceptin (trastuzumab), used in breast cancer, is directed to the 30% of breast cancers with an overexpression of the HER-2 protein, which responds to Herceptin. Gleevec (Imatinib mesylate) is used to treat chronic myeloid leukemia, which has increased life expectancy from 5% to 95% at five years. Zelboraf (Vemurafenib) is used to treat melanoma, where the late-stage prognosis has been dismal, but 60% of patients have a defect in their DNA, and this drug benefits those with the V600E defect. Other successful personalized medicine examples of “treatment–biomarker” combinations are in colon cancer (Erbitux–EFGR) and lung cancer (Xalkori–ALK) [76].

Two other fields of active work are *de novo* drug design [77] and the optimization of gene therapies [78]. Drug discovery is a very challenging problem due to the high attrition rates in *de novo* design due to the lack of the efficacity of the new compounds and due to possible development of undesirable side effects [79,80,81]. The computational problem consists of finding a new compound that provides the desirable structure–activity relationships (SAR data) [82,83]. This is a very challenging problem due to the high dimensionality of the databases to explore the chemical space, which can be cast as an optimization and/or sampling problem. Local optimization approaches and deep learning methodologies can deal with such problems, but they are unable to perform a complete sampling of the chemical space due to the curse of the dimensionality problem. Additionally, local optimization methods might converge to suboptimal solutions that might be far away from the global solution.

Gene therapy is an experimental technique that uses genes to treat or prevent disease [84]. Several approaches to gene therapy are tested:

1. Replacing a mutated gene that causes disease with a healthy copy of the gene.
2. Inactivating, or “knocking out,” a mutated gene that is functioning improperly.
3. Introducing a new gene into the body to help fight a disease.

This promising treatment technique remains risky and requires computational methods to understand the effect of these therapies on gene expression and on proteomics, and how they can affect the health of the patients.

## 6. Data Mining Tools/Algorithms and Their Applications for Personalized Medicine

The machine learning algorithms are significant to interpret the genomic datasets and help in the design of personalized medicines. The use of multimodal data helps in a deeper analysis of large datasets, which improves the understanding of human health and disease by leaps and bound. Algorithms represent the terminal node in the final predictions from big data [85]. Lee and coauthors proposed a person-centered data mining algorithm that could simultaneously integrate both genetic information and baseline profiles to identify which individual person will benefit from a specific antipsychotic drug among schizophrenic patients. The proposed algorithm can be easily adopted in many other clinical practices for personalized medicine [86]. To analyze metagenomes from novel environmental niches, Ulyantsev and coauthors developed an algorithm named “MetaFast”, which enabled them to compare the microflora of a healthy person with the microflora of a patient. As a result, specialists would be able to detect previously unidentified pathogens and their strains, which can aid in the development of personalized medicine [87]. Furthermore, it must be emphasized that the idea behind algorithms is not to replace physicians, but to provide them with tools that support their decisions based on the wealth of available biomedical knowledge and data-driven criteria.

### 6.1. Pattern-Based Approaches in Data Mining for Analyzing Patient Data

Pattern mining concentrates on identifying rules that describe specific patterns within the data. Pattern mining is the discovery of sequential patterns, for example, sequences of errors or warnings that precede equipment failure may be used to schedule preventative maintenance or may provide insight into a design flaw. Genomic and medical studies have continuously been collecting a huge amount of data on a daily basis and analysis of these data is becoming a challenge with every passing day. Analyzing this huge amount of data needs some practical approaches to deal with it. Sequence data analysis approaches provide several different ways to uncover the precious hidden knowledge in data bulks and to discover novel or important patterns related to a particular disease or individual patient. Here, in this section, we will discuss some of pattern-based approaches (e.g., clustering and temporal pattern analysis) commonly utilized for data mining to analyze the patient’s data. Temporal data are more often related to clinical studies and mainly depend on time series, with or without a sequence of events (i.e., the time-based quantitative measurements or sequence of temporal events related to particular clinical study) [88].

### 6.2. Network Mining for Personalized Medicine and Health Care

To discover the meaningful patterns, interactions, relations, and clinical rules among the variants, data mining and machine learning methods are used to build models for systems biology. Data mining is the “process of sorting through large datasets to identify patterns and establish relationships to solve problems through data analysis”. The medical industry collects a dazzling array of data, most of which are electronic health records (EHRs) collected by HIPAA covered health care facilities. According to a survey by PubMed, data mining is becoming increasingly popular in health care, if not increasingly essential. The huge amounts of data generated by health care EDI transactions cannot be processed and analyzed using traditional methods because of the complexity and volume of the data. Data mining methods include artificial neural networks, clustering, Bayesian networks, decision trees, and genetic algorithms. However, to classify different variants according to the classifications defined by biomedical experts, machine learning techniques are useful, and multiple drug targets could be found by using these techniques. Similar to clustering, the expression of big data at the level of genes and proteins can help in the identification of biomarkers and target candidates [9]. In personalized medicine, medical records representing the very personal biomedical information (individually identifiable health information) are guarded very carefully by the Health Insurance Portability and Accountability Act (HIPAA) and are not available openly. These types of data are not shared centrally to prevent the misuse of big data methods. Furthermore, medical records store the standard medical and clinical data gathered from the patients. There are many errors such as altered data quality and misinterpretations, improper grammatical use, spelling errors, local dialects, and semantic ambiguities, which increase the complexity of data processing and analysis for medical records. Therefore, there is a strong need for the data preprocessing of medical records such as data cleansing, data integration, data transformation, data reduction, and privacy protection [9].

### 6.3. Big Data Management Problems in Precision Medicine and Health Care

Different types of barriers including philosophical, legal, and practical exist that cause hindrance in the access to data. To improve the translation of big data to health care solutions, several issues need to be addressed that include collecting and standardizing the heterogenous data; data curation; data de-identification and anonymization; legal consents that are required for using the available data; and the importance of providing that data back to the health care communities for further research and usage. As the volumes of big data generated are increasing exponentially, the complexity of these data increases. Sequencing individual human genome is no longer enough, as transcript-level expression analyses of RNA-Seq experiments, metabolites, proteome data, phenotypic and functional traits, can now also be associated within the data. Moreover, earlier research has shown that significant information from a single cell data can provide more details about the biological processes in comparison to the bulk analysis of multiple cell types in a mixed cell population [89]. There are new ways for measuring the single cell genome and transcriptome sequencing (G&T-seq) allowing us to simultaneously obtain both transcriptomic and genomic information from a single cell [90], which can provide clearer information that may be helpful in designing precision medicine [9]. Data volumes are increasing continuously and beyond comprehension, while there is a shortage of bioinformaticians in the current scenario [91]. Prior knowledge about the related domains is considerably helpful to build models based on the big data, so it is suggested, ideally, that analysts should also be trained in biomedicine in addition to bioinformatics. Translational research is usually focused on collecting data abundantly, for example, clinical data, imaging data, genetic and genomic data, and analysts are usually found to be lacking in skills to interpret such types of data [10,62,92].

### 6.4. Significance of Next Generation Informatics for Big Data in Precision Medicine Era

These approaches can help to transform biomedical data into useful drug development information, and to apply the knowledge for decision support in clinical practice. Today, data integration techniques related to the field of biomedical sciences and health care settings are rapidly revolutionizing research domains by acting as a bridge between biological and medical sciences and data mining. This certainly rests upon a record data upsurge in biological knowledge and research. In the modern era, the field of bioinformatics is facing a challenging task to handle and interpret the massive amounts of genomics, proteomics, and metabolomics data, which are accumulating at an unprecedentedly fast pace. Sparse, noisy, and discontinuous data need special care, which is difficult using traditional machine learning and existing computational methods. Numerous promising solutions have been exploited to tackle big data mining problems and provide creative solutions. In this sense, we must consider that there is no single solution for any biomedical problem. As we have stated in the conceptualization of big data section, there could be infinite models that solve a biomedical problem due to the huge uncertainty space [93]. One of the possible approaches to deal with this ill-posed problem is to sample the uncertainty space using a wide multeity of models. There is no unique model capable of solving this problem perfectly, so we need to explore different techniques, obtaining a solution with its uncertainty assessment, using a consensus strategy [94]. This way, we could give robust information to a medical expert to enhance the medical decision process. There are multiple applications of this methodology in the field of proteomics [95], genomics [96], clinical prognosis [97], cancer treatment [98], aging [99], analysis of defective pathways [100,101], and drug repositioning [102].

The idea is to benefit from biomedical data and apply resourceful informatics approaches to reform the practice of medicine and to improve the health care system. Implementing these approaches promises a bright era of next generation precision medicine [59]. Research strategies that facilitate up-to-date all-encompassing biomedical expertise along with handling vast health care data are highly required.

## 7. Heterogeneity, a Huge Challenge in Big Data Analysis

**Figure 3:**
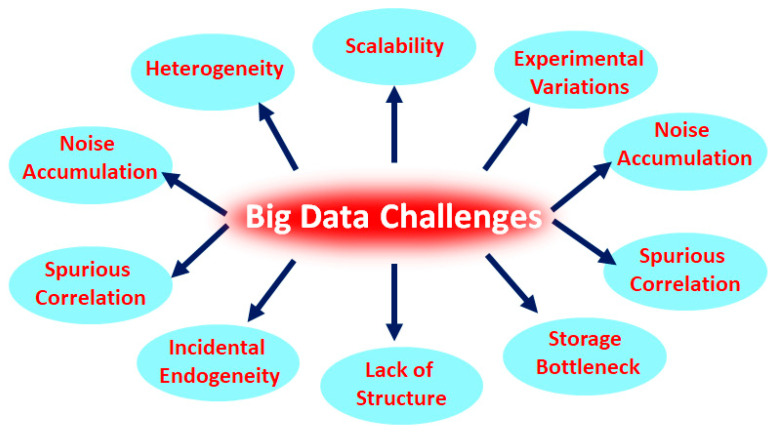
Big data challenges in recent times.

Although big data analysis promises great advantages and a potential solution to a diverse range of problems, there remain many unique technical, computational, and statistical challenges that must be addressed to fully explore its potential [103]. Heterogeneity [104], incompleteness, complexity, privacy problems, scalability, lack of structure, storage bottlenecks, spurious correlations, incidental endogeneity, noise accumulation, experimental variations, statistical biases, and measurement errors impede progress at all phases of the big data analysis from data collection and analysis to result in elucidation that can create value from the data [105,106]. In order to solve this issue, data structuring should be the first key step in, or prior, to data analysis. Let us consider a patient with several medical procedures at a hospital, where one record per entire hospital stay or medical procedure or laboratory test can relatively ease the problem of heterogeneity. The value of data explodes when it can be linked with other data, thus data integration is a major creator of value. Moreover, heterogeneity might be terminological, conceptual, syntactic, or semiotic in nature [104,107]. The problems start right away during data collection as decisions have to be made concerning what data to keep and what to discard, and how to store what is kept reliably. Cloud computing, along with more sophisticated statistical methods, can provide a solution as it offers high scalability, reliability, and autonomy, along with composability and dynamic resource discovery. Moreover, federated learning is also a machine learning method that enables machine learning models to obtain experience from different datasets located at different sites (e.g., local data centers, a central server) without sharing the training data. This allows for personal data to remain in local sites, reducing the possibility of personal data breaches. To handle these challenges, we require transformative solutions, therefore, it is time to develop advanced statistical and computational methods that are robust to data complexity, noises, and data dependence (Figure 3) [103].

## 8. Role of Big Data in Accelerating Digital Healthcare

Big data analysis will empower digital health care in a way that will ensure the timely access of clinicians to the entire scope of a patient’s health information while reducing the need for in-person visits and improving patient outcome [108]. Through digital medicine, big data analysis will prove to be an invaluable tool for health care organizations as it will provide more opportunities for proactive intervention and a more holistic view of the patients’ conditions with consolidated real-time information [109]. The health care providers’ ability to connect with health care apps to track and monitor patient health will be another key benefit. In addition, via risk modeling and stratification, big data analysis will help to make more accurate predictions of where a patient’s health is trending. By utilizing data-driven insights into patient health, big data analyses will eventually be beneficial for the patients themselves, especially for those who can utilize telemedicine and remote patient monitoring to their advantage, in order to enjoy more flexible and convenient access to care, which in turn will help them to live healthier lives [110,111,112].

## 9. Big Data Applications in Health Care

There are so many applications where big data is being implemented to enhance patient care, and, ultimately, can save lives [113]. There are two major divisions of health care big data: vital and social data. The vital category is more significant compared to social big data. However, social big data can also be significant to the health care industry by allowing practitioners to detect attitudes through sentiment analysis [114].

## 10. Electronic Health Records

Electronic health records (EHRs) are most significant application of big data in medicine and health care [115,116]. The patients must have their medical records reporting demographics, medical history, allergies, and laboratory test results in digital forms. Every record is comprised of one modifiable file, which means that doctors can implement changes over time with no paperwork and no danger of data replication [117].

## 11. Health Big Data as a Key Player for Informed Strategic Planning

Strategic planning through big data analytics improves the understanding of people’s motivations. A common practice is to analyze the check-up results among people in different demographic groups and identify which factors discourage people from taking up treatment. Therefore, better understating of these data and better strategic plans can cure more patients in the most diverse areas [118].

## 12. Advanced Risk and Disease Management through Big Data

Another significant use of big data is essential for tackling the hospitalization risk for particular patients with chronic diseases [119,120]. More precisely, it can also be used to prevent deterioration. Moreover, by drilling down into insights such as medication type, symptoms, and the frequency of medical visits, among many others, it is possible for health care institutions to provide accurate preventative care and, ultimately, reduce hospital admissions [121].

## 13. Developing New Therapies and Big Data

Big data in the health care department has the power to discover new medications [122,123,124]. By using prior data record, real-time, predictive metrics, and cohesive mix data visualization techniques, health care experts can identify the potential strengths and weaknesses in clinical trials or therapeutic processes. Moreover, through data-driven analysis of genetic information as well as efficient predictions for patients, big data analytics in health care can play an important role in the development of new ground breaking drugs and innovative, forward-thinking therapies [125]. Data analytics in health care can streamline, innovate, provide security, save lives, and give confidence and clarity [126].

## 14. Impediments of Big Data in Health Care

One of the major problems in the use of big data in medicine is that medical data have been collected across different states, hospitals, and administrative departments using different protocols [127,128,129]. Therefore, new infrastructure resources are required to better cross-examine the medical data through proper collaboration between different data providers. A newly designed software with better efficacy is required for the health care department. We will move from standard regression-based methods, which are a subset of supervised machine learning methods, because the standard regression models can be used in the machine learning framework to learn from the data and provide outcome predictions based on the inputs [130].

It is known that during online data collection and transmission, health care devices are functional with the help of their MAC addresses and Internet protocol (IP) addresses [97,131]. However, there are serious problem related to the use of this procedure because these network addresses can be accessed and linked to the location and the name of the device’s owner, so by analyzing the transmitted data, a hacker could identify an individual including financial and other confidential information. Moreover, there are various open software applications that can track cell phone locations and the names of social media users through MAC and GPS big data, which might be used for malicious reasons [132,133]. Based on such problems, most countries have created legislative principles to secure the personal health care privacy and confidentiality of medical records such as the HIPAA under the Privacy Rule of 2003 in the United States [134,135].

## 15. Conclusions and Future Prospects

The big data paradigm shift is significantly transforming health care and biomedical research [109,136,137,138,139], having the potential to better process clinical and biomolecular information that spans the four dimensions of volume, velocity, variety, and veracity, referring to scale, rate, forms, and content of generated data, respectively [140]. In the current situation, large amount of genomics data could be used to address personalized health care issues [104] and can help to propose new drugs for the treatment of gene related disorders. Advanced machine learning approaches such as artificial intelligence and deep learning represent the future toolbox for the data-driven analytics of genomic big data. The emerging progress in these areas will be indispensable for future innovation in health care and personalized medicine.

## Data Availability

Not applicable.
